# Larval Exposure to the Bacterial Insecticide *Bti* Enhances Dengue Virus Susceptibility of Adult *Aedes aegypti* Mosquitoes

**DOI:** 10.3390/insects9040193

**Published:** 2018-12-14

**Authors:** Isabelle Moltini-Conclois, Renaud Stalinski, Guillaume Tetreau, Laurence Després, Louis Lambrechts

**Affiliations:** 1 Insect-Virus Interactions Group, Department of Genomes and Genetics, Institut Pasteur, 75015 Paris, France; isabelle.conclois@gmail.com; 2 Centre National de la Recherche Scientifique, Génomique Evolutive, Modélisation et Santé, UMR 2000, 75015 Paris, France; 3 Laboratoire d’Ecologie Alpine, Université Grenoble Alpes, Centre National de la Recherche Scientifique, UMR 5553, 38058 Grenoble, France; renaud.stalinski@gmail.com (R.S.); laurence.despres@univ-grenoble-alpes.fr (L.D.); 4 Institut de Biologie Structurale, Université Grenoble Alpes, Commissariat à l’Energie Atomique, Centre National de la Recherche Scientifique, UMR 5075, 38058 Grenoble, France; guillaume.tetreau@gmail.com

**Keywords:** *Bacillus thuringiensis* subsp. *israelensis*, carry-over effect, arbovirus, vector competence, multiple infection

## Abstract

Understanding the interactions between pathogens sharing the same host can be complicated for holometabolous animals when larval and adult stages are exposed to distinct pathogens. In medically important insect vectors, the effect of pathogen exposure at the larval stage may influence susceptibility to human pathogens at the adult stage. We addressed this hypothesis in the mosquito *Aedes aegypti*, a major vector of arthropod-borne viruses (arboviruses), such as the dengue virus (DENV) and the chikungunya virus (CHIKV). We experimentally assessed the consequences of sub-lethal exposure to the bacterial pathogen *Bacillus thuringiensis* subsp. *israelensis* (*Bti*), during larval development, on arbovirus susceptibility at the adult stage in three *Ae. aegypti* strains that differ in their genetic resistance to *Bti*. We found that larval exposure to *Bti* significantly increased DENV susceptibility, but not CHIKV susceptibility, in the *Bti*-resistant strains. However, there was no major difference in the baseline arbovirus susceptibility between the *Bti*-resistant strains and their *Bti*-susceptible parental strain. Although the generality of our results remains to be tested with additional arbovirus strains, this study supports the idea that the outcome of an infection by a pathogen depends on other pathogens sharing the same host even when they do not affect the same life stage of the host. Our findings may also have implications for *Bti* as a mosquito biocontrol agent, indicating that the sub-optimal *Bti* efficacy may have counter-productive effects by increasing vector competence, at least for some combinations of arbovirus and mosquito strains.

## 1. Introduction

Although host–pathogen interactions are often studied as a one-to-one relationship, most hosts are simultaneously or sequentially infected with several pathogens [1]. Because different infections are not necessarily independent from each other, infection probability and dynamics should be evaluated in the context of pathogen communities [2,3]. Pathogens sharing the same host may interact directly through competition for resources, or indirectly via the host immune response [4,5].

Understanding interactions between pathogens infecting the same host can be complicated for holometabolous animals (i.e., with complete metamorphosis) when larval and adult stages live in separate habitats and are exposed to distinct pathogens. Larval traits can have multiple, complex fitness consequences that persist across the metamorphic boundary [6]. In mosquito vectors of human pathogens, for instance, conditions experienced at the larval stage can influence adult traits underlying vectorial capacity, such as pathogen susceptibility and lifespan [7,8,9].

Here, we investigated whether such a ‘carry-over’ effect of exposure to a larval pathogen influences susceptibility to another pathogen at the adult stage. We used the mosquito *Aedes aegypti*, a major vector of several medically significant arthropod-borne viruses (arboviruses). Mosquitoes are holometabolous insects whose larvae develop in water prior to their metamorphosis into flying adults. We examined the consequences of a sub-lethal exposure to the bacterial pathogen *Bacillus thuringiensis* subsp. *israelensis* (*Bti*) during larval development on susceptibility to the dengue virus (DENV) and the chikungunya virus (CHIKV) at the adult stage.

*Bti* is a natural mosquito pathogen known for its environmental safety and used as a biological control agent [10]. *Bti* is currently used worldwide as an alternative to chemical insecticides, particularly in Europe where it is the main insecticide used against mosquito larvae outdoors. During sporulation, *Bti* produces four main toxins: Cry4Aa, Cry4Ba, Cry11Aa and Cyt1Aa. Upon ingestion by larvae, the toxins form pores in the midgut epithelium, allowing invasion by *Bti* and gut bacteria and resulting in larval death [11].

*Aedes* species and populations vary in their natural susceptibility to *Bti* [12,13]. High levels of resistance to each individual Cry toxin of *Bti* can evolve in only a few generations of selection in the laboratory, but they only confer a moderate overall resistance to *Bti* [14,15]. Genetic resistance to *Bti* is associated with constitutive changes in the expression of immune genes [16,17]. Mosquito life-history traits (survival, development time, fecundity) were found to respond to *Bti* selection, suggesting that *Bti*-resistance alleles may have pleiotropic effects throughout the mosquito life cycle [18].

In the present study, we hypothesized that immunological or physiological changes in mosquitoes exposed to *Bti* toxins at immature stages might affect virus–mosquito interactions and thus arbovirus susceptibility at the adult stage. To account for the genetic differences in *Bti* susceptibility, we compared the consequences of sub-lethal *Bti* exposure in three different *Ae. aegypti* strains with different levels of *Bti* resistance. The Bora-Bora strain is naturally susceptible to *Bti*, and the LR4A and LR3Bti strains were obtained by artificial selection of the Bora-Bora strain for Cry4Aa and *Bti* resistance, respectively [15].

## 2. Materials and Methods

### 2.1. Mosquitoes

The three *Ae. aegypti* strains used in this study (Bora-Bora, LR4A, LR3Bti) have been described elsewhere [15]. Briefly, mosquitoes of the *Bti*-susceptible Bora-Bora strain were selected during 33 generations for resistance to one of the three Cry toxins (Cry4Aa, Cry4Ba, Cry11Aa). The LR4A strain is 1018-fold more resistant to Cry4Aa than the parental Bora-Bora strain, but displays little cross-resistance to the other Cry toxins (<4-fold cross-resistance to Cry4Ba and Cry11Aa). The composite LR3Bti strain was initially created by mixing 30% of each of the three resistant strains (i.e., selected for resistance to individual *Bti* toxins) and 10% of the Bora-Bora strain, followed by 14 generations of selection for *Bti* resistance [19]. The LR3Bti strain is 5- to 14-fold more resistant to individual *Bti* toxins and 1.4-fold more resistant to *Bti* than the Bora-Bora strain. The mosquitoes were reared under standard insectary conditions (27 °C, 70% relative humidity and a 12 h light:12 h dark cycle) as previously described [20].

### 2.2. Bti Exposure

Eggs from each of the three strains were hatched under reduced air pressure for 1 h. Because the LR4A and LR3Bti strains develop slower than the Bora-Bora strain, their eggs were hatched 24 h earlier to synchronize adult emergence. After hatching, 200 first-instar larvae from each strain were sorted into 24 × 34 × 9 cm plastic trays filled with 1.5 L of dechlorinated tap water. The larvae were fed on a standard diet of Tetramin fish food (Tetra) for 4–5 days. Groups of 100 third-instar larvae from each strain were placed into Coplin jars containing 30 mg of fish food (Tetra). They were exposed (treatment) or non-exposed (control) to a dose of *Bti* (toxins and spores) adjusted to reach the 50% lethal concentration (LC_50_) of each strain (151.9 μg/mL for the Bora-Bora strain and 216.2 μg/mL for the LR4A and LR3Bti strains). After 24 h of exposure to *Bti*, the surviving fourth-instar larvae were returned to their rearing trays. The number of larval rearing trays was 6, 9 and 12 for the Bora-Bora, LR4A and LR3Bti strains, respectively. The number of Coplin jars for *Bti* exposure was 12, 18 and 24 for the Bora-Bora, LR4A and LR3Bti strains, respectively. The pupae were transferred to cages and the emerging adults were maintained under standard insectary conditions with permanent access to 10% sucrose solution.

### 2.3. Arbovirus Exposure

Following *Bti* exposure at the larval stage, 5- to 8-day-old adult females were exposed to the dengue virus (DENV; type 1 strain KDH0030A [21]) or the chikungunya virus (CHIKV; strain 06-21 [22]) in an artificial infectious blood meal as previously described [20]. For each treatment and arbovirus, the adults were exposed to the infectious blood meal in 1, 2–3 and 2 boxes of 60 females for the Bora-Bora, LR4A and LR3Bti strains, respectively. The blood feeding rate was 51.7% on average and ranged from 25.8% to 68.3% between the boxes. The rabbit blood draws performed in the context of this study were approved by the Institutional Animal Care and Use Committee at Institut Pasteur, under protocol number 2015-0032. The virus stocks were prepared in C6/36 (*Aedes albopictus*) cells and titrated by fluorescent focus-forming assay (FFA) as previously described [20]. The final blood meal titer was 4.75 × 10^5^ focus-forming units (FFU)/mL for DENV and 5.38 × 10^5^ FFU/mL for CHIKV.

### 2.4. Arbovirus Susceptibility

Susceptibility to DENV and CHIKV was measured by (*i*) the proportion of females that became infected (infection rate) and (*ii*) the proportion of infected mosquitoes in which the virus disseminated to the head tissues (dissemination rate). After 7 days (CHIKV) and 10 days (DENV) of incubation, mosquitoes were harvested and dissected. The heads were separated from their bodies with a clean scalpel that was decontaminated between each individual with Hexanios G + R 0.5% (Anios). The two time points were chosen to reflect the difference in the viral dissemination kinetics between CHIKV and DENV [23]. The infection rates were measured by the detection of viral RNA in the mosquito bodies. The dissemination rates were measured by detection of the infectious virus in the head tissues of body-positive mosquitoes. The detection of DENV RNA in the body samples was performed by reverse transcription (RT) quantitative PCR as previously described [20]. The detection of CHIKV RNA in the body samples was performed by RT-PCR as follows. The total RNA was reverse-transcribed to complementary DNA (cDNA) with random hexamers. The cDNA was amplified by PCR (forward primer: 5′-AAGCTYCGCGTCCTTTACCAAG-3′; reverse primer: 5′-CCAAATTGTCCYGGTCTTCCT-3′). The amplicons were visualized by electrophoresis on 2% agarose gels. For both DENV and CHIKV, the heads were homogenized individually and processed for infectious virus detection using a qualitative version of the FFA [20] that only tests the undiluted sample and therefore does not allow end-point titration.

### 2.5. Bti Persistence

To measure *Bti* persistence at the adult stage, 4-day-old adult males and females from the LR4A and LR3Bti strains that survived 24 h of *Bti* exposure (LC_50_) at the third-instar larval stage were homogenized in 200 µL PBS. In order to determine whether *Bti* could persist as spores and/or as vegetative cells throughout mosquito development, the homogenates were divided into two parts. One half was heated for 60 min at 80 °C, in order to kill most bacteria except the *Bti* spores that survive this treatment [24]. Both the heated and non-heated homogenates were plated separately on nutrient agar. After 24 h at 30 °C, the bacterial colonies were identified and counted.

### 2.6. Statistical Analyses

The proportion of infected mosquitoes and the proportion of infected mosquitoes with a disseminated infection were analyzed as binary response variables (Table S1) using logistic regression. The treatment (*Bti* vs. unexposed) and strain (LR3Bti, LR4A, Bora-Bora) were included as covariates in a full-factorial model. The pairwise differences were assessed by likelihood-ratio **χ**^2^ tests, followed by Bonferroni correction for multiple testing. All analyses were performed in JMP v10 (SAS Institute Inc., Cary, NC, USA).

## 3. Results

**Figure 1:**
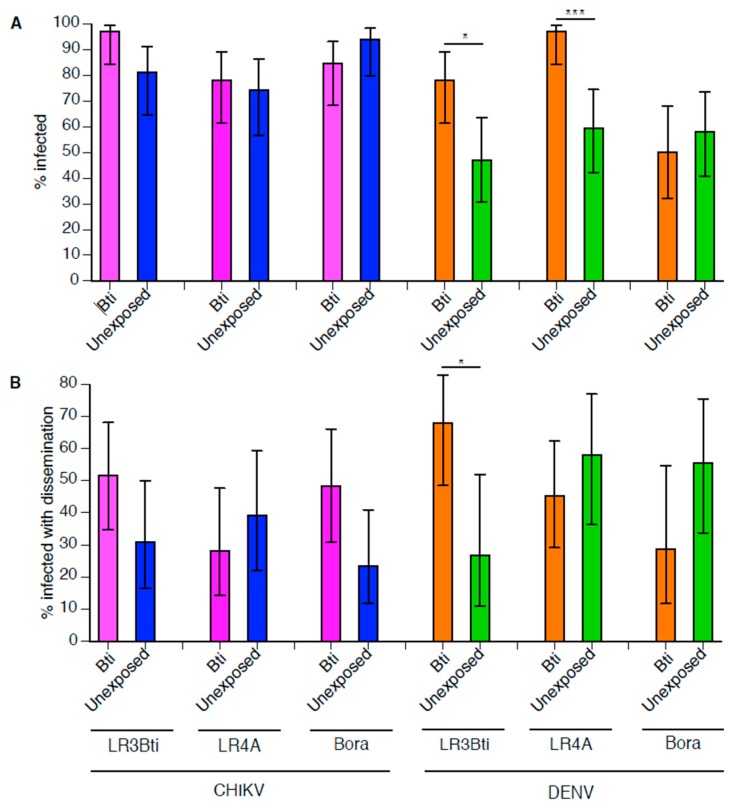
Arbovirus susceptibility following sub-lethal *Bacillus thuringiensis* subsp. *israelensis* (*Bti)* exposure during larval development. For each arbovirus, the chikungunya virus (CHIKV) and the dengue virus (DENV), the percentage of infected mosquitoes (**A**) and the percentage of infected mosquitoes with a disseminated infection (**B**) are shown as a function of strain and treatment. Error bars represent 95% confidence intervals of the percentages. Stars above the bars indicate statistical significance of pairwise differences after Bonferroni correction for multiple testing (* *p* < 0.05; ** *p* < 0.01; *** *p* < 0.001). Test statistics for the full model are provided in Table 1.

Following sub-lethal *Bti* exposure at the larval stage, a total of 184 (57–64 per strain) *Ae. aegypti* females were orally challenged with DENV. Overall, 65.8% of the mosquitoes became infected with DENV and 49.6% of the infected mosquitoes developed a disseminated infection. DENV infection was influenced by a strong interaction between the strain and the treatment (Table 1). In pairwise comparisons, DENV infection was significantly higher in the *Bti*-exposed mosquitoes of the LR3Bti (*p* = 0.027) and LR4A (*p* = 0.0006) strains relative to the unexposed controls, whereas there was no effect of *Bti* exposure in the Bora-Bora strain (Figure 1). The DENV dissemination rate was also influenced by a strong interaction between the strain and the treatment (Table 1). In pairwise comparisons, DENV dissemination was significantly higher in the *Bti*-exposed mosquitoes of the LR3Bti strain (*p* = 0.0303), but there was no effect of *Bti* exposure in the LR4A and Bora-Bora strains (Figure 1).

Following sub-lethal *Bti* exposure at the larval stage, a total of 191 (63–64 per strain) *Ae. aegypti* females were orally challenged with CHIKV. Overall, 84.8% of the mosquitoes became infected with CHIKV and 37.0% of the infected mosquitoes developed a disseminated infection. The CHIKV infection rate was only marginally significantly influenced by the strain (Table 1), due to a slightly lower infection rate of LR4A mosquitoes, irrespective of the *Bti* treatment. There was no significant effect of the *Bti* treatment or interaction between the strain and the treatment on the CHIKV infection rates (Table 1; Figure 1). The CHIKV dissemination rate was not significantly influenced by the strain, the treatment, or their interaction (Table 1; Figure 1).

A total of 18 adults from the LR4A (*n* = 3) and LR3Bti (*n* = 15) strains were tested for *Bti* persistence at the adult stage. None of them were found positive for *Bti*.

## 4. Discussion

We tested whether sub-lethal pathogen exposure at the larval stage would influence pathogen susceptibility at the adult stage in a holometabolous insect. We found evidence supporting this hypothesis in the mosquito vector *Ae. aegypti*, although it depended on the mosquito strain and the infecting pathogen. Exposure to the bacterial pathogen *Bti* at the larval stage subsequently enhanced adult susceptibility to DENV, but not CHIKV, and only in the *Bti*-resistant strains, not in the *Bti*-susceptible strain. However, there was no evidence for an association between the level of genetic resistance to *Bti* and intrinsic DENV or CHIKV susceptibility. Note that the lack of significant effect for CHIKV in our experiment could be due to the higher overall infection rate across the strains and treatments (84.8%) than for DENV (65.8%). This may have reduced our power to detect an increase in the CHIKV infection rates between the strains and treatments.

Although these results will need to be followed up with additional experiments using different arbovirus strains [25], they support the idea that the outcome of host infection by a given pathogen depends on other pathogens sharing the same host [2,4]. They go one step further by showing that this can be the case even when pathogens do not affect the same life stage. Although the underlying mechanism is unknown, the observed carry-over effect is unlikely to result from direct competition because *Bti* did not persist at the adult stage when the mosquitoes were orally challenged with the DENV. We speculate that it more likely results from indirect effects, such as tissue damage, immune impairment, or reserve depletion.

Our results differ from a previous study reporting that following *Bti* exposure at the larval stage, *Ae. aegypti* females had similar rates of DENV infection and dissemination than unexposed controls [7]. This discrepancy could be due to several factors. Firstly, we used laboratory strains of *Ae. aegypti,* whereas the earlier study used a field-derived population. We observed that the response to *Bti* exposure differed among *Ae. aegypti* strains, indicating that the effect of *Bti* varies across mosquito genotypes. Secondly, we exposed third-instar larvae to their *Bti* LC_50_, whereas in the earlier study first-instar larvae were exposed to concentrations of *Bti* that were 4- to 24-fold lower than their LC_50_. Thirdly, the effect could be dependent on the DENV strain, which was not the same between the studies.

Our protocol eliminated 50% of individuals at the larval stage and therefore the carry-over effect that we observed could be confounded with natural selection. In other words, non-random elimination of 50% of the individuals could contribute to explaining differences in DENV susceptibility that are not a direct consequence of sub-lethal *Bti* exposure, but rather are due to the selection of genetically more-susceptible individuals. However, because the *Bti*-resistant strains were selected for at least 14 generations prior to this experiment [15], it is unlikely that one additional generation of selection would have substantially changed the genetic composition of these strains.

Although the *Bti*-resistant strains exhibit both different gene expression profiles and specific polymorphisms relative to their *Bti*-susceptible parental strain [26], we did not observe major differences in the baseline arbovirus susceptibility (in the absence of *Bti* exposure) between the LR4A, LR3Bti and Bora-Bora strains. However, the *Bti*-resistant strains displayed higher DENV susceptibility at the adult stage after *Bti* exposure at the larval stage, suggesting that *Bti*-selected genetic changes may have contributed to the carry-over effect. In addition to putative Cry-toxins receptors, many genes involved in the cuticle/chitin metabolism and in gut enzymatic functions (e.g., detoxification, trans-membrane transport, proteolytic activities) were constitutively differently expressed in the *Bti*-resistant strains relative to the Bora-Bora strain [17,26]. Interestingly, two specific antimicrobial peptides were over-expressed in the Bora-Bora strain upon *Bti* exposure [15], however this response has not been investigated in the *Bti*-resistant strains. Another important consequence of *Ae. aegypti* exposure to *Bti* is a dramatic change in the composition of the larval gut bacterial microbiome [27], which could have mediated the carry-over effect on DENV susceptibility [8].

Finally, our findings have potential implications for *Bti* as a mosquito biocontrol agent. Exposure to a sub-lethal dose of *Bti* increased DENV susceptibility in the surviving adults of the *Bti*-resistant strains, potentially canceling out the larvicidal effect by increasing permissiveness to arbovirus infection in the surviving individuals. Although other parameters, such as adult mosquito density and survival, have to be taken into account to quantitatively estimate the net effect of *Bti* on vectorial capacity [7,8], our results indicate that the sub-optimal *Bti* efficacy may have counter-productive effects by increasing vector competence.

## 5. Conclusions

Although the generality of our results remains to be tested with additional arbovirus strains, this study supports the idea that the outcome of an infection by a pathogen depends on other pathogens sharing the same host even when they do not affect the same life stage of the host. Our results also have implications for *Bti* as a mosquito biocontrol agent, because they indicate that *Bti* may have counter-productive effects on vectorial capacity when its efficacy is sub-optimal.

## Figures and Tables

**Table 1:** Test statistics of arbovirus susceptibility phenotypes.

		Infection		Dissemination	
	df	LR χ^2^	*p* Value	LR χ^2^	*p* Value
**DENV**					
Strain	2	13.5	0.0012	0.57	0.7527
Treatment	1	12.5	0.0004	0.03	0.8562
Strain × Treatment	2	11.8	0.0027	9.25	0.0098
**CHIKV**					
Strain	2	6.82	0.0331	0.69	0.7068
Treatment	1	0.60	0.4401	2.17	0.1404
Strain × Treatment	2	5.58	0.0613	4.26	0.1187

df = degrees of freedom; LR = likelihood ratio.

## Supplementary Materials

The following are available online at http://www.mdpi.com/2075-4450/9/4/193/s1: Table S1. Raw dataset. For each strain, treatment and arbovirus, the susceptibility phenotypes are indicated (0 = uninfected; 1 = infected).
