# Supplementary material for: Larval Exposure to the Bacterial Insecticide Bti Enhances Dengue Virus Susceptibility of Adult Aedes aegypti Mosquitoes
Source: Insects. 2018 Dec 14;9(4):193. doi: 10.3390/insects9040193 (PMC6316598; doi:10.3390/insects9040193)
Supplement: Supplementary file 1 [file insects-09-00193-s001.pdf]

Strain	Treatment	Virus	Infection	Dissemination
Bora	Control	CHIKV	1	0
Bora	Control	CHIKV	1	0
Bora	Control	CHIKV	1	0
Bora	Control	CHIKV	1	0
Bora	Control	CHIKV	1	1
Bora	Control	CHIKV	1	0
Bora	Control	CHIKV	1	1
Bora	Control	CHIKV	1	0
Bora	Control	CHIKV	1	0
Bora	Control	CHIKV	1	0
Bora	Control	CHIKV	1	0
Bora	Control	CHIKV	1	0
Bora	Control	CHIKV	1	0
Bora	Control	CHIKV	1	0
Bora	Control	CHIKV	1	0
Bora	Control	CHIKV	1	0
Bora	Control	CHIKV	1	1
Bora	Control	CHIKV	1	1
Bora	Control	CHIKV	1	0
Bora	Control	CHIKV	1	1
Bora	Control	CHIKV	1	0
Bora	Control	CHIKV	1	0
Bora	Control	CHIKV	1	0
Bora	Control	CHIKV	0	0
Bora	Control	CHIKV	1	0
Bora	Control	CHIKV	1	0
Bora	Control	CHIKV	1	0
Bora	Control	CHIKV	1	1
Bora	Control	CHIKV	1	1
Bora	Control	CHIKV	0	0
Bora	Control	CHIKV	1	0
Bora	Control	CHIKV	1	0
Bora	Bti	CHIKV	1	1
Bora	Bti	CHIKV	0	0
Bora	Bti	CHIKV	1	0
Bora	Bti	CHIKV	1	0
Bora	Bti	CHIKV	1	0
Bora	Bti	CHIKV	1	1
Bora	Bti	CHIKV	1	0
Bora	Bti	CHIKV	0	0
Bora	Bti	CHIKV	1	0
Bora	Bti	CHIKV	1	0
Bora	Bti	CHIKV	1	1
Bora	Bti	CHIKV	1	0
Bora	Bti	CHIKV	1	0
Bora	Bti	CHIKV	1	1
Bora	Bti	CHIKV	1	1
Bora	Bti	CHIKV	1	1
Bora	Bti	CHIKV	0	0
Bora	Bti	CHIKV	1	0
Bora	Bti	CHIKV	1	1
Bora	Bti	CHIKV	1	1
Bora	Bti	CHIKV	0	0
Bora	Bti	CHIKV	1	1
Bora	Bti	CHIKV	1	1
Bora	Bti	CHIKV	1	0
Bora	Bti	CHIKV	1	0
Bora	Bti	CHIKV	1	0
Bora	Bti	CHIKV	1	0
Bora	Bti	CHIKV	1	1
Bora	Bti	CHIKV	0	0
Bora	Bti	CHIKV	1	1
Bora	Bti	CHIKV	1	1
Bora	Bti	CHIKV	1	0
LR4A	Control	CHIKV	1	0
LR4A	Control	CHIKV	1	1
LR4A	Control	CHIKV	1	1

LR4A	Control	CHIKV	1	1
LR4A	Control	CHIKV	1	0
LR4A	Control	CHIKV	1	0
LR4A	Control	CHIKV	1	1
LR4A	Control	CHIKV	1	1
LR4A	Control	CHIKV	1	1
LR4A	Control	CHIKV	1	1
LR4A	Control	CHIKV	1	0
LR4A	Control	CHIKV	1	0
LR4A	Control	CHIKV	1	1
LR4A	Control	CHIKV	1	0
LR4A	Control	CHIKV	1	0
LR4A	Control	CHIKV	0	0
LR4A	Control	CHIKV	0	0
LR4A	Control	CHIKV	0	0
LR4A	Control	CHIKV	0	0
LR4A	Control	CHIKV	1	0
LR4A	Control	CHIKV	1	0
LR4A	Control	CHIKV	1	0
LR4A	Control	CHIKV	1	0
LR4A	Control	CHIKV	0	0
LR4A	Control	CHIKV	1	0
LR4A	Control	CHIKV	0	0
LR4A	Control	CHIKV	0	0
LR4A	Control	CHIKV	1	1
LR4A	Control	CHIKV	1	0
LR4A	Control	CHIKV	1	0
LR4A	Control	CHIKV	0	0
LR4A	Bti	CHIKV	1	1
LR4A	Bti	CHIKV	1	1
LR4A	Bti	CHIKV	0	0
LR4A	Bti	CHIKV	1	0
LR4A	Bti	CHIKV	1	0
LR4A	Bti	CHIKV	1	0
LR4A	Bti	CHIKV	1	0
LR4A	Bti	CHIKV	0	0
LR4A	Bti	CHIKV	1	1
LR4A	Bti	CHIKV	1	0
LR4A	Bti	CHIKV	0	0
LR4A	Bti	CHIKV	1	0
LR4A	Bti	CHIKV	1	0
LR4A	Bti	CHIKV	0	0
LR4A	Bti	CHIKV	1	0
LR4A	Bti	CHIKV	1	0
LR4A	Bti	CHIKV	1	0
LR4A	Bti	CHIKV	1	1
LR4A	Bti	CHIKV	1	1
LR4A	Bti	CHIKV	1	0
LR4A	Bti	CHIKV	1	0
LR4A	Bti	CHIKV	1	0
LR4A	Bti	CHIKV	0	0
LR4A	Bti	CHIKV	1	0
LR4A	Bti	CHIKV	0	0
LR4A	Bti	CHIKV	1	1
LR4A	Bti	CHIKV	1	0
LR4A	Bti	CHIKV	1	0
LR4A	Bti	CHIKV	0	0
LR4A	Bti	CHIKV	1	0
LR4A	Bti	CHIKV	1	0
LR4A	Bti	CHIKV	1	1
LR3Bti	Control	CHIKV	1	0
LR3Bti	Control	CHIKV	1	1
LR3Bti	Control	CHIKV	1	1
LR3Bti	Control	CHIKV	0	0
LR3Bti	Control	CHIKV	1	0
LR3Bti	Control	CHIKV	1	0
LR3Bti	Control	CHIKV	1	0
LR3Bti	Control	CHIKV	1	0

LR3Bti	Control	CHIKV	1	0
LR3Bti	Control	CHIKV	1	0
LR3Bti	Control	CHIKV	1	0
LR3Bti	Control	CHIKV	0	0
LR3Bti	Control	CHIKV	0	0
LR3Bti	Control	CHIKV	1	0
LR3Bti	Control	CHIKV	1	0
LR3Bti	Control	CHIKV	0	0
LR3Bti	Control	CHIKV	1	0
LR3Bti	Control	CHIKV	1	1
LR3Bti	Control	CHIKV	1	0
LR3Bti	Control	CHIKV	1	1
LR3Bti	Control	CHIKV	1	0
LR3Bti	Control	CHIKV	1	1
LR3Bti	Control	CHIKV	1	0
LR3Bti	Control	CHIKV	1	0
LR3Bti	Control	CHIKV	1	0
LR3Bti	Control	CHIKV	1	0
LR3Bti	Control	CHIKV	0	0
LR3Bti	Control	CHIKV	1	1
LR3Bti	Control	CHIKV	1	0
LR3Bti	Control	CHIKV	1	1
LR3Bti	Control	CHIKV	1	1
LR3Bti	Control	CHIKV	0	0
LR3Bti	Bti	CHIKV	0	0
LR3Bti	Bti	CHIKV	1	1
LR3Bti	Bti	CHIKV	1	1
LR3Bti	Bti	CHIKV	1	1
LR3Bti	Bti	CHIKV	1	0
LR3Bti	Bti	CHIKV	1	1
LR3Bti	Bti	CHIKV	1	0
LR3Bti	Bti	CHIKV	1	0
LR3Bti	Bti	CHIKV	1	0
LR3Bti	Bti	CHIKV	1	1
LR3Bti	Bti	CHIKV	1	1
LR3Bti	Bti	CHIKV	1	1
LR3Bti	Bti	CHIKV	1	0
LR3Bti	Bti	CHIKV	1	0
LR3Bti	Bti	CHIKV	1	1
LR3Bti	Bti	CHIKV	1	1
LR3Bti	Bti	CHIKV	1	0
LR3Bti	Bti	CHIKV	1	0
LR3Bti	Bti	CHIKV	1	0
LR3Bti	Bti	CHIKV	1	1
LR3Bti	Bti	CHIKV	1	1
LR3Bti	Bti	CHIKV	1	1
LR3Bti	Bti	CHIKV	1	1
LR3Bti	Bti	CHIKV	1	0
LR3Bti	Bti	CHIKV	1	0
LR3Bti	Bti	CHIKV	1	0
LR3Bti	Bti	CHIKV	1	0
LR3Bti	Bti	CHIKV	1	0
LR3Bti	Bti	CHIKV	1	1
LR3Bti	Bti	CHIKV	1	0
LR3Bti	Bti	CHIKV	1	1
LR3Bti	Bti	CHIKV	1	1
Bora	Control	DENV	1	1
Bora	Control	DENV	0	0
Bora	Control	DENV	0	0
Bora	Control	DENV	1	1
Bora	Control	DENV	1	1
Bora	Control	DENV	1	0
Bora	Control	DENV	0	0
Bora	Control	DENV	0	0
Bora	Control	DENV	1	0
Bora	Control	DENV	1	0
Bora	Control	DENV	1	0
Bora	Control	DENV	1	1

Bora	Control	DENV	0	0
Bora	Control	DENV	0	0
Bora	Control	DENV	1	1
Bora	Control	DENV	1	1
Bora	Control	DENV	1	1
Bora	Control	DENV	1	0
Bora	Control	DENV	1	1
Bora	Control	DENV	0	0
Bora	Control	DENV	0	0
Bora	Control	DENV	0	0
Bora	Control	DENV	0	0
Bora	Control	DENV	1	1
Bora	Control	DENV	0	0
Bora	Control	DENV	1	1
Bora	Control	DENV	0	0
Bora	Control	DENV	1	0
Bora	Control	DENV	1	0
Bora	Control	DENV	1	0
Bora	Control	DENV	0	0
Bora	Bti	DENV	0	0
Bora	Bti	DENV	0	0
Bora	Bti	DENV	0	0
Bora	Bti	DENV	1	0
Bora	Bti	DENV	1	0
Bora	Bti	DENV	0	0
Bora	Bti	DENV	1	1
Bora	Bti	DENV	0	0
Bora	Bti	DENV	1	0
Bora	Bti	DENV	0	0
Bora	Bti	DENV	1	0
Bora	Bti	DENV	0	0
Bora	Bti	DENV	0	0
Bora	Bti	DENV	0	0
Bora	Bti	DENV	0	0
Bora	Bti	DENV	0	0
Bora	Bti	DENV	0	0
Bora	Bti	DENV	1	0
Bora	Bti	DENV	0	0
Bora	Bti	DENV	1	1
Bora	Bti	DENV	1	1
Bora	Bti	DENV	1	0
Bora	Bti	DENV	1	0
Bora	Bti	DENV	1	1
Bora	Bti	DENV	1	0
Bora	Bti	DENV	1	0
LR4A	Control	DENV	1	0
LR4A	Control	DENV	1	1
LR4A	Control	DENV	1	1
LR4A	Control	DENV	1	1
LR4A	Control	DENV	1	1
LR4A	Control	DENV	1	1
LR4A	Control	DENV	1	1
LR4A	Control	DENV	0	0
LR4A	Control	DENV	1	1
LR4A	Control	DENV	0	0
LR4A	Control	DENV	0	0
LR4A	Control	DENV	0	0
LR4A	Control	DENV	1	0
LR4A	Control	DENV	0	0
LR4A	Control	DENV	1	1
LR4A	Control	DENV	1	1
LR4A	Control	DENV	1	1
LR4A	Control	DENV	1	0
LR4A	Control	DENV	1	0
LR4A	Control	DENV	1	0
LR4A	Control	DENV	1	0
LR4A	Control	DENV	1	0
LR4A	Control	DENV	0	0

LR4A	Control	DENV	0	0
LR4A	Control	DENV	1	0
LR4A	Control	DENV	0	0
LR4A	Control	DENV	0	0
LR4A	Control	DENV	0	0
LR4A	Control	DENV	0	0
LR4A	Control	DENV	0	0
LR4A	Control	DENV	1	1
LR4A	Bti	DENV	1	1
LR4A	Bti	DENV	1	1
LR4A	Bti	DENV	1	0
LR4A	Bti	DENV	1	1
LR4A	Bti	DENV	1	0
LR4A	Bti	DENV	1	1
LR4A	Bti	DENV	1	0
LR4A	Bti	DENV	1	0
LR4A	Bti	DENV	1	1
LR4A	Bti	DENV	1	1
LR4A	Bti	DENV	1	0
LR4A	Bti	DENV	1	0
LR4A	Bti	DENV	1	0
LR4A	Bti	DENV	1	1
LR4A	Bti	DENV	1	1
LR4A	Bti	DENV	1	1
LR4A	Bti	DENV	1	0
LR4A	Bti	DENV	1	0
LR4A	Bti	DENV	1	0
LR4A	Bti	DENV	1	1
LR4A	Bti	DENV	1	0
LR4A	Bti	DENV	1	1
LR4A	Bti	DENV	1	0
LR4A	Bti	DENV	1	0
LR4A	Bti	DENV	1	0
LR4A	Bti	DENV	1	1
LR4A	Bti	DENV	1	0
LR4A	Bti	DENV	1	1
LR4A	Bti	DENV	0	0
LR4A	Bti	DENV	1	0
LR4A	Bti	DENV	1	0
LR4A	Bti	DENV	1	1
LR3Bti	Control	DENV	0	0
LR3Bti	Control	DENV	0	0
LR3Bti	Control	DENV	0	0
LR3Bti	Control	DENV	1	1
LR3Bti	Control	DENV	1	1
LR3Bti	Control	DENV	0	0
LR3Bti	Control	DENV	1	1
LR3Bti	Control	DENV	1	0
LR3Bti	Control	DENV	0	0
LR3Bti	Control	DENV	0	0
LR3Bti	Control	DENV	0	0
LR3Bti	Control	DENV	0	0
LR3Bti	Control	DENV	1	0
LR3Bti	Control	DENV	1	0
LR3Bti	Control	DENV	0	0
LR3Bti	Control	DENV	0	0
LR3Bti	Control	DENV	0	0
LR3Bti	Control	DENV	1	0
LR3Bti	Control	DENV	1	0
LR3Bti	Control	DENV	1	0
LR3Bti	Control	DENV	0	0
LR3Bti	Control	DENV	0	0
LR3Bti	Control	DENV	0	0
LR3Bti	Control	DENV	1	0
LR3Bti	Control	DENV	1	0
LR3Bti	Control	DENV	0	0
LR3Bti	Control	DENV	0	0
LR3Bti	Control	DENV	0	0

LR3Bti	Control	DENV	1	1
LR3Bti	Control	DENV	1	0
LR3Bti	Control	DENV	1	0
LR3Bti	Control	DENV	1	0
LR3Bti	Bti	DENV	0	0
LR3Bti	Bti	DENV	1	1
LR3Bti	Bti	DENV	1	1
LR3Bti	Bti	DENV	1	1
LR3Bti	Bti	DENV	1	1
LR3Bti	Bti	DENV	1	1
LR3Bti	Bti	DENV	1	0
LR3Bti	Bti	DENV	1	1
LR3Bti	Bti	DENV	0	0
LR3Bti	Bti	DENV	1	1
LR3Bti	Bti	DENV	1	1
LR3Bti	Bti	DENV	1	1
LR3Bti	Bti	DENV	1	0
LR3Bti	Bti	DENV	1	0
LR3Bti	Bti	DENV	0	0
LR3Bti	Bti	DENV	0	0
LR3Bti	Bti	DENV	1	1
LR3Bti	Bti	DENV	1	1
LR3Bti	Bti	DENV	0	0
LR3Bti	Bti	DENV	1	0
LR3Bti	Bti	DENV	1	0
LR3Bti	Bti	DENV	1	0
LR3Bti	Bti	DENV	0	0
LR3Bti	Bti	DENV	1	1
LR3Bti	Bti	DENV	1	0
LR3Bti	Bti	DENV	1	1
LR3Bti	Bti	DENV	1	1
LR3Bti	Bti	DENV	1	1
LR3Bti	Bti	DENV	1	1
LR3Bti	Bti	DENV	1	0
LR3Bti	Bti	DENV	0	0
LR3Bti	Bti	DENV	1	1
